# Advances in Extraction Methods to Recover Added-Value Compounds from Seaweeds: Sustainability and Functionality

**DOI:** 10.3390/foods10030516

**Published:** 2021-03-02

**Authors:** Gabriela S. Matos, Sara G. Pereira, Zlatina A. Genisheva, Ana Maria Gomes, José A. Teixeira, Cristina M. R. Rocha

**Affiliations:** 1 CEB—Centre of Biological Engineering, Campus Gualtar, University of Minho, 4710-057 Braga, Portugal; gabriela.souza@ceb.uminho.pt (G.S.M.); sarapereira@ceb.uminho.pt (S.G.P.); zlatina@ceb.uminho.pt (Z.A.G.); jateixeira@deb.uminho.pt (J.A.T.); 2 Centro de Biotecnologia e Química Fina—Escola Superior de Biotecnologia, Universidade Católica Portuguesa/Porto, 4169-005 Porto, Portugal; amgomes@porto.ucp.pt

**Keywords:** seaweeds, added-value compounds, sustainable technologies, biorefinery, food applications

## Abstract

Seaweeds are a renewable natural source of valuable macro and micronutrients that have attracted the attention of the scientists in the last years. Their medicinal properties were already recognized in the ancient traditional Chinese medicine, but only recently there has been a considerable increase in the study of these organisms in attempts to demonstrate their health benefits. The extraction process and conditions to be used for the obtention of value-added compounds from seaweeds depends mainly on the desired final product. Thermochemical conversion of seaweeds, using high temperatures and solvents (including water), to obtain high-value products with more potential applications continues to be an industrial practice, frequently with adverse impact on the environment and products’ functionality. However more recently, alternative methods and approaches have been suggested, searching not only to improve the process performance, but also to be less harmful for the environment. A biorefinery approach display a valuable idea of solving economic and environmental drawbacks, enabling less residues production close to the much recommended zero waste system. The aim of this work is to report about the new developed methods of seaweeds extractions and the potential application of the components extracted.

## 1. Introduction

Seaweeds or macroalgae are multicellular photosynthetic plant-like organisms that grow mainly in the seas and oceans. They represent an economically valuable natural resource, widely available and their potential as food-grade feedstock should not be disregarded, particularly in the forecast context of nutrients shortage due to the global population growth. Further, seaweeds sustainable cultivation and harvesting is feasible, as sea water covers ca. 70% of the Earth surface and they have generally high growth rates, do not compete with agriculture for land and potable water and have low or neutral greenhouse gas emissions. Thus, mariculture has the potential to supply environmentally sustainable and healthy foods, ensuring that the United Nations Sustainable Development Goals and Paris Agreement are achieved, making them widely accessible to different layers of society [1].

Countries in East and Southeast of Asia are dominant both in seaweed farming and consumption. The world production of marine macroalgae has more than tripled from 10.6 to 32.4 millions of tons in the period between 2000 and 2016 [2]. In the last decade, more than 291 species have been used as food, feed, fertilizer or for industrial applications [3]. Even though nowadays seaweeds are not yet normally included within western diets, the main use of seaweed worldwide is for direct human consumption. Seaweeds are also used directly in animal feed or in soils as natural fertilizers [4], but the most relevant application, besides food, is in the production of ingredients (mainly, but not limited to, hydrocolloids) for the food and cosmetic industries.

However, the real nutrition potential varies from species to species, seaweeds have generally been identified as a rich source of valuable macro and micronutrients like proteins, polyphenols, pigments, minerals as well as carbohydrates such as carrageenan and alginate. Moreover, some species are also rich in vitamins, such as C and B_12_ [5]. Seaweeds are traditionally used in Asia for their nutritional properties, as well as for therapeutic applications. However, only in recent years, there has been a considerable increase in the study of bioactivities and bioprospecting of these organisms in attempts to enlighten their health effects and related mechanisms [6].

Though seaweeds may be consumed directly, the industrial focus is on the thermal conversion of seaweeds to obtain added-value products with more potential applications than the conventional raw material [7]. The current processes have several limitations for industries, mostly focused on the high consumption of energy, solvent and time. Furthermore, severe processes, with high temperatures and very long extraction times, may cause deleterious effects on the compounds and their functionalities. Lately, other than thermochemical process methods are used for the extraction of seaweeds. The new developed methods search not only to improve the process, but also to be less harmful for the environment.

An attractive option for the seaweed valorization is to use the concept of a biorefinery [8]. The extraction of a single product is frequently not profitable, as it can involve high costs and generate large amounts of wastes, whereas a biorefinery process aims to fully maximize the use of the biomass to produce multiple products, with minimal waste generation.

In this context, this review will focus on the latest improvements in the methods used for the extraction of bioactive compounds from seaweeds and their sustainable application into value-added products with different functionalities.

## 2. Seaweeds Classification and Chemical Composition

The group of macroalgae, also known as seaweeds, is composed by autotrophic and photosynthetic organisms that can measure from few centimeters to 100 m of length. Different from terrestrial plants, they do not present conductive tissues, absorbing nutrients on all their exposed surfaces [9]. The seaweeds can be classified based on their pigmentation into three groups: brown seaweeds (phylum Ochrophyta, class Phaeophyceae), red seaweed (phylum Rhodophyta) and green seaweed (phylum Chlorophyta) [10]. More than 16,000 species are described. The red macroalgae is the biggest group containing about 7300 known species, followed by the green and brown macroalgae with 6700 and 2000 identified species, respectively [11]. The taxonomic classification becomes complex due to the different criteria applied. According to the kingdoms of life proposed by Cavalier-Smith [12], Rhodophyta and Chlorophyta are included in the kingdom Plantae, while Ochrophyta is in the kingdom Chromista.

The red seaweeds are dominated by the presence of phycoerythrin and phycocyanin pigments, covering the expression of other colors produced by chlorophyll *a*, *b*-carotene and xanthophylls [13]. The green seaweeds are rich in chlorophyll *a* and *b* pigments, which hide the appearance of *b*-carotene and xanthophylls also present [13], varying their colors from greenish yellow to dark green. Green seaweeds present high growth and reproduction rates, irrespective of geographical location and season, enabling their production at large-scale and harvest to be more than once in a year. The brown seaweeds have a pigmentation that varies from yellow to dark brown. These colors appear because of the xanthophyll pigment fucoxanthin’s high concentration, masking other pigments such as *b*-carotene, chlorophyll *a* and *c*, as well as other xanthophylls [13]. This group of seaweed is divided into two subgroups: kelps, such as *Laminaria hyperborean*, *Laminaria ochroleuca* and *Saccorhiza polyschides*, which can achieve more than 50 m in height and create subaquatic forests; and fucales, such as *Fucus vesiculosus*, *Fucus serratus* and *Himanthalia elongate* (known as sea spaghetti) [10].

Marine seaweeds have an interesting nutritional profile, being a rich source of biocompounds belonging to different chemical groups like polysaccharides, proteins, minerals, pigments, phenolic compounds and lipids. The biochemical composition of seaweeds depends on several environmental and stress conditions such as salinity, cultivation location, depth, changes in temperature, nutrient enrichment, harvesting period, UV radiation exposure, intensity of herbivores or pollutants and biotic factors such as species, life stage, age or reproductive status [14,15,16]. All these factors can influence the production of secondary metabolites that have shown a wide range of biological properties, such as: anti-inflammatory, antioxidant, antimicrobial, anti-proliferative, antiviral, antidiabetic, anticancer, neuroprotective and photoprotective [4,17,18,19,20,21,22,23,24]. Others external aspects that may have effects on the seaweeds profile include the different procedures of extraction and pretreatments used to obtain the bioactive compounds. The wide chemical composition of seaweeds, their diversity and availability, allows a huge potential for different applications [25]. For example, the technological attributes of hydrocolloids, such as alginate, carrageenan and agar, make them the most regularly used gelling compounds by industry for foods, pharmaceutical and biotechnological applications [26].

### 2.1. Carbohydrates

Polysaccharides are mainly located in the cell walls of seaweeds. The polysaccharides content in seaweeds is species dependent with values ranging from 4% to 76% dry weight (d.w.) [27], with a wide application due to their functional and bioactive properties [28]. Their chemical composition and molecular weight are also dependent on the species of the raw seaweed as well as on the extraction procedure. It is well known that different groups of seaweeds contain different compounds. Brown seaweeds (Phaeophyceae) have high content of polysaccharides as laminarian, fucoidan, and alginate. Seaweeds of the phyla Chlorophyta and Rhodophyta (green and red seaweeds) contain other groups of polysaccharides, such as ulvan, agar and carrageenan [29]. The polysaccharides carrageenan and agar (with important thickening and gelling features) are mainly found in red seaweed and ulvan in green seaweed [13,30].

Generally, brown seaweeds contain high amounts of soluble carbohydrates and are known to produce three major polysaccharides: alginates, laminarins and fucoidans [23]. The physiological and biological properties of fucoidans are very interesting to the pharmaceutical industry, due to their anticoagulant, antioxidant, antiviral, antimicrobial, anti-tumor, anti-mutagenic, immune-modulating, and anti-inflammatory activities [31,32,33]. Laminarins have also demonstrated good bioactive properties such as antibacterial, antioxidative and anticoagulant potential. Due to their resistance to hydrolysis in the upper gastrointestinal tract, they are classified as dietary fibers [28]. Alginate provides to the seaweed flexibility and stability against the marine currents. Such mechanical feature can be used in industry as thickener, stabilizer or/and gelling agent, important to the food, cosmetic or biomedical sectors [34]. It is estimated that commercial brown seaweeds may contain up to 40% of its d.w. as sodium alginate. Further, mannitol is the main chemical compound found in four brown seaweeds (i.e., *Carpophyllum exuosum*, *Carpophyllum plumosum*, *Ecklonia radiata*, *Undaria pinnatifida*) accounting for between 22.2–30.7% d.w. [8] and in the context of biorefinery, mannitol itself is considered an added-value by-product.

Due to the high content of carbon and low content of lignin, macroalga have been considered a viable biomass for biofuels production and are included in the third generation bioethanol resources [35,36]. However, as several fractions of macroalgae have high added value, bioethanol production is usually considered as a part of a biorefinery process where high added value fractions are recovered for other applications and only the residual low value fraction, usually rich in cellulose, is used as substrate, after hydrolysis, in the fermentative bioethanol production process.

Seaweed polysaccharides are also used in agriculture, as they stimulate plant growth or protect them from diseases since they can inhibit plant viruses and prevent fungal diseases and bacterial infection [29].

### 2.2. Proteins

Depending on the species and the season, the protein content of seaweed varies between 5% and 47% d.w. [27], covering all the essential amino acids. Moreover, some species of seaweed (mainly from red seaweeds) are known to contain protein levels similar to those of traditional protein sources, such as meat, egg, soybean, and milk [30]. In this context seaweed is a viable alternative source of protein. Brown seaweeds usually contain a low protein content (up to 15%) when compared to that of green (up to 26%) or red seaweed (up to 47%). However, protein bioavailability in seaweeds is usually low, due to their higher content of polysaccharides and dietary fiber [37,38].

Study on the amino acid composition of seaweeds demonstrated that green seaweeds have lower percentages of aspartic and glutamic acids, the percentages of lysine and arginine were higher in red seaweeds, while brown seaweeds tended to show more methionine than green and red seaweeds [39]. Seaweeds are a source of lysine, an essential amino acid often present in low quantities in terrestrial plant-protein sources, and in contrast to animal-protein sources, are not associated with cardiovascular diseases and diabetes, and so have been highly recommend for a healthy and balance diet [38]. Furthermore, species such as *Ulva* and *Caulerpa* contain high levels of arginine and glycine, in addition to histidine and taurine which present important activities for fetus development [40].

### 2.3. Pigments

The main natural pigments found in marine seaweed are chlorophylls, carotenoids and phycobiliproteins. Chlorophyll is the primary pigment in macroalgae that is responsible for the energy caption from the sunlight, while other pigments like carotenoids, also produced by seaweeds, assist in transporting the adsorbed energy and in the protection of tissues against photo oxidative damage [8]. Carotenoids are known to have positive influence on human nutrition and health, and the best known biological function of carotenoids is the provitamin A activity, responsible for the ability to form vitamin A [41].

Phycobiliproteins are the principal photoreceptor for photosynthesis in red seaweeds [42], being phycoerythrin (red color) the most abundant [42,43]. The pigments in brown seaweeds were found to be between 3.4–9.8% of the algal dry weight, and the predominant pigment compounds were pheophytin *a*, a derivative of chlorophyll [8] and the carotenoid fucoxanthin [42]. Like other compounds, the pigment content varies with the season, such as in *Ascophyllum*, their content of violaxanthin, fucoxanthin, carotene and chlorophyll *a* were higher from March to June and lowest from July to November [44].

The main pigments present in green macroalgae belong to the groups of chlorophylls (chlorophylls *a* and *b*) and carotenoids (xanthophylls) [45]. For example, in *Ulva lactuca* L. was detected in diminishing order: chlorophyll *a*, chlorophyll *b*, 9-cis-carotene, *b*-carotene, trans *b*-carotene, carotene-isomers, violaxanthin, astazanthein, antheraxanthin, lutein, zeaxantin and cryptoxanthin [46].

### 2.4. Phenolic Compounds

Phenolic compounds are secondary metabolites that play an important role in the development and growth of plants. They are also synthetized in macroalgae and their total phenolic content (TPC) has been positively correlated with their corresponding antioxidant activity [47]. The group of brown seaweeds has been reported to have the highest amounts of phenolic compounds [48]. Further, brown seaweeds contain a specific group of phenolic compounds called phlorotannins. Inside the seaweed cell, the phlorotannins can be found in the soluble or insoluble form [49]. E.g., eighteen different phlorotannins were found in brown seaweed from the Romanian Black Sea coast [21]. Meanwhile, in green and red seaweeds the phenolic compounds found in largest proportion are bromophenols, flavonoids, phenolic acids, phenolic terpenoids and mycosporine-like amino acids [15].

### 2.5. Minerals

Mineral content of seaweeds is generally high and varies from 8% to 40% [50]. The main minerals found in seaweeds are P, Na, K, Ca, Mg, Fe, Cu, Zn and Mn. The mineral concentration depends on the type of seaweed i.e., brown, green or red. Brown seaweeds have higher concentrations of K and Ca compared to green and red macroalgae while green seaweeds have higher content in Mg, Fe and Cu [51]. Moreover, the mineral content of seaweed varies according to the season [50,52]. Their ability of being bioadsorptive and bioaccumulative, makes the mineral content from 10 to 100 times higher than in land plants [53].

### 2.6. Lipids

As for the other types of compounds, the total lipid content of seaweeds is affected by light, temperature, atmospheric constituents, different geographical locations and time of collection [52,54]. Further, seaweed extracts’ lipid composition and extraction yield are strongly influenced by the solvents used [55]. However, in general, the lipid content in seaweeds is low (<7% DW). Despite the low content of lipids, these are rich in omega-3 polyunsaturated fatty acids, making this an interesting seaweed’s fraction [53]. For instance, some red seaweeds contain docosahexaenoic acid (DHA) which is recognized as one of the most important n-3 polyunsaturated fatty acid (PUFA) to reduce the risk of cardiovascular diseases [56].

### 2.7. Vitamins

Some seaweeds contain about a dozen times more vitamins than terrestrial plants or animal, including fat and water soluble vitamins such as A, D, E, K, C, B_1_, B_2_, B_9_ and B_12_, with emphasis on the last one for being one of the few found in a non-animal source and present in active form that can be absorbed by humans; this is important to vegetarian diets [1,26,57]. Red seaweeds are an important source of vitamin A, and species such as *Porphyra umbilicalis* and *Gracilaria changii* have high quantities of vitamin C [15]. According to [58] the seasonality is an important factor, reporting that the highest content of vitamin C was found in seaweeds collected in the Northern Hemisphere around April and May.

## 3. Recovery of Added-Value Compounds

The fractionation processes for the recovery of seaweed functional biocompounds can be divided in: raw material pre-treatment, extraction (single or multiple steps), followed by separation and concentration [28]. In many cases the use of additional pre-treatments, such as alkali pretreatment to remove sulphate groups from agar, improve the recovery final results [49,59]. The current processes have several limitations for industries, mostly focused on the high consumption of energy, solvent and time. Moreover, severe processes, with high temperatures and very long extraction times, may cause deleterious effects to the compounds and their functionalities, which underlies the importance to develop and apply new methods of extractions.

### 3.1. Conventional Extraction Methods

Conventional solid-liquid extractions, depending on the seaweed species and the type of biocompounds, often require many hours of extraction to accomplish optimum extraction yield and quality [60]. These may include infusion, percolation, Soxhlet extraction, maceration, steam distillation, among others [28,60,61,62]. The solvent choice is an important step for the solubilization of the target compound, allowing selectivity, high efficiency with the increase of mass transfer, tissue damage, solvent diffusion, in combination with different extraction technologies or stirring [62]. For instance, in the case of phycocolloids, the most popular solvent is hot water due to the hydrocolloids’ water solubility and because it is a major component of fresh biomass [60,62]. Hot alkali extraction (also aqueous-based) is used for alginate/alginic acid extraction, converting insoluble alginate salts into water soluble alginate salts. Depending on the target compound other solvents like acids or organic solvents (hexane, chloroform, methanol, ethanol, etc.) are commonly used.

The heating mechanism of conventional extractions is also critical for a successful extraction and usually involves convection through the solvent and conduction from the surfaces to the nucleus of the matrix particles [63]. Further, it is difficult to optimize the bioactive compounds extraction since they are highly dependent on harvesting season, seaweed sources, geographical region, concentration of solvents, temperature, time, pH and raw material size [60].

Nowadays, industrial practices employ large amounts of chemical solvents during pre-treatment and extraction, generating effluents throughout the production process, which can be health and environment hazardous [60]. Regarding these common weaknesses of conventional extractions, other methods have been approached, from an economic perspective, called “green” or “alternative” methods, which may reduce energy, solvents and extraction time to a few minutes. The development of sustainable extraction methods is a key to boost value-addition of the globally expanding seaweed industry [28].

### 3.2. Alternative Extraction Methods

As mentioned above, conventional extractions use severe and harsh processing conditions, which can generate large amounts of hazardous wastes and raise environmental concerns, as the creation of a substantial carbon footprint. Moreover, if the used solvent is toxic, the downstream processing is essential to accomplish the food and pharmaceutical industry regulations for application of safe end-products [28]. In this context, sustainable extraction methods are currently a popular hot topic for research and development in the recovery of natural products [28,60]. Further, the use of eco-friendly solvents, such as ionic liquids, eutectic solvents, surfactants or solvents produced from biomass, is another strategy to diminish the negative impact of the extraction process. An example is the use of edible oils for the extraction of carotenoids from brown seaweeds [41]. The definition of smart technologies is attributed to technologies that have the following features: non-chemical, fast, energy-efficient, zero waste, non-hazardous, eco-friendly, low cost, versatile, applicable to untreated biomass and able to combine with other green technologies [62]. Nowadays, for industry, the main aspects are quality, economy and eco-friendliness. The green extraction methods search to reduce or avoid the use of solvents and increase the extraction yields [28,60,62]. Several factors have to be taken into consideration when selecting the extraction method: cost, time, recovery rate, volume and type of solvents, “greenness” of the method and a possibility of scale-up. The conventional and less eco-friendly extraction methods for seaweed biocompounds are the most common around the world. However, it is essential to demonstrate through continuous research, the efficiency and safety of the sustainable methods, in order to reduce implementation costs and captivate investment for industrial implementation [28,60]. Table 1 presents an overview of the main alternative technologies that have been applied in the extraction of different biocompounds from seaweeds and the yields achieved.

#### 3.2.1. Microwave-Assisted Extraction

Microwave-assisted extraction (MAE) is the most used, cost-effective and non-chemical technology nowadays at the laboratory scale. This technology uses microwave irradiation, generating directly heat within the matrix, through collisions and frictions between molecules, and, consequently, resulting in fast heating (a few seconds) [60,62,63]. In other words, MAE operates in the electromagnetic waves spectrum, which waves are non-ionizing and the frequency range between 300 MHz and 300 GHz. The waves’ absorption can originate two different processes, dipole rotation and ionic conduction, depending on temperature and causing structural changes within the matrix. Dipole rotation is based on dipolar fractions alignment of the samples with the electric field created by the microwaves, colliding among themselves and generating heat. Ionic conduction is based on the movement of charged ions due to the electrostatic field influence created by the microwaves, and the friction of these movements creates heat [28,62,63]. Important factors to consider include frequency, power, extraction time, extraction pressure, ratio of solid/liquid, solvent concentration, solvent properties, matrix characteristics, temperature and number of extraction cycles.

MAE has many advantages, such as the consistency, efficiency, capability of heating raw materials selectively and locally, improved mass transfer and tissue disruption, less extraction time, less energy and solvent consumption, low cost, high extraction rate and high quality of products. MAE can be applied directly in fresh biomass, and is currently used in the extraction of different biocompounds (i.e., polysaccharides, phenolics, etc.) from brown, red and green seaweeds [28,60,62]. Magnusson et al. [76] showed that MAE could increase 70% of polyphenols yields from *Carpophyllum flexuosum* compared to conventional extraction and Boulho et al. had 20% of carrageenan from *Solieria chordalis* [77]. However, due to the fast heat generation of this technology and difficulties in accurately controlling temperature, it is quite difficult to extract some thermolabile compounds (i.e., fatty acids, pigments, proteins) [28]. There are two types of systems: the open and closed systems. The closed system is used at higher pressure and temperature, and there is a possible risk of explosion. The open system is cheaper, fully automatic, and used under atmospheric pressure, which overcomes the problem of an explosion. However, it is less precise, not able to work with simultaneous samples and requires longer extraction time when compared to closed systems [60,63].

MAE can be scaled up to the industrial level, though with important restrictions due to the limited wave penetration into the feedstock matrices. Furthermore, a significant initial capital is needed and the equipment costs and operator skills are high, so more research is required [28,60].

#### 3.2.2. Ultrasound-Assisted Extraction

Ultrasound-assisted extraction (UAE) is defined by sound waves with high frequencies >20 KHz, that propagate in compression and rarefaction waves. The passage of an ultrasound wave induces the effect of acoustic cavitation in the solvent and the formation of cavitation bubbles, which increase the contact surface area between solid and liquid phase. This allows a better solvent penetration into the matrix and a fast solute diffusion. Asymmetrical bubbles are generated by solid-liquid suspensions, the vapor from the solvent gets entrapped within the bubble, leading to implosion and generating mechanical energy through microturbulence, which break the algal cell wall and enhance extraction efficiency [28,60,62,63]. Parameters that may influence the extraction process include pressure, temperature, intensity and frequency of the waves, surface tension and viscosity of the solvent [28]. UAE improves the extraction yield and reduces extraction time. It is an efficient, eco-friendly, simple and not expensive extraction technology in comparison to other methods. It has been applied in the food industry due to its low cost of equipment maintenance, small equipment size, direct scale-up to industrial scale and decreased number of process steps [28,60,62]. There are still a small number of studies on the extraction of compounds, mainly referring to phenolics, proteins and polysaccharides from marine seaweeds using UAE, though with very promising results [28,60,62]. Rafiquzzaman et al. [81] showed that carrageenan yield and purity were better with UAE using alkaline and aqueous solvent than conventional extraction. Kadam et al. [72] also concluded that laminarin extraction using UAE had greater yield and antioxidant activity when comparing to conventional methods [60]. The major drawback is the fact that UAE needs a significant starting capital for industrial scale due to the energy input and equipment cost and its use is still limited. However, it can be an interesting technology used alone or in combination with others methods [28,60,62].

#### 3.2.3. Enzyme-Assisted Extraction

Enzyme-assisted extraction (EAE) is a technology that requires food digestive enzymes, such as proteases and carbohydrases to break down the complex seaweeds cell wall and isolate the active compounds. This sustainable extraction method can be considered the greenest among all the methods, as it does not use any harmful chemicals or organic solvents. There are important parameters that improve the extraction efficiency of targeted biocompounds, including pH, temperature, type of solvent (water or buffer), ratio substrates:solvent:enzymes and mixing conditions [60,63]. EAE has several advantages, such as eco-friendly, low cost, conversion of water-insoluble materials into water-soluble materials, high efficiency and specificity in target end-products, process scalability and reduced extraction time [28,61,63]. Up to date, there are few studies of EAE application in seaweeds. EAE was used for carrageenan extraction from red seaweeds achieving high extraction yield and good gelling properties [60]. Other studies showed that EAE preserved the structural integrity of the target compounds (i.e., fucoidans, ulvans, proteins, lipids, etc.). Moreover, the obtained products had important bioactivities suitable for cosmetic, nutraceutical and pharmaceutical industries [28,61,73]. For instance, significant antihypertensive activity has been described for hydrolyzed peptides from red seaweeds [86,87]. EAE extraction applied on *Sargassum muticum*, *Osmundea pinnatifida*, and *Codium tomentosum* presented the highest nitrogen and total phenolics contents, highest sulfated polysaccharide contents and highest prebiotic activity, respectively. *O. pinnatifida* and *C. tomentosum* EAE extracts showed inhibitory potential against α-glucosidase (38–49%) [86].

There are very few available enzymes for industrial use, specifically for substrates found in seaweeds, and the ones that are available still have prohibitive costs. Therefore, most EAE applications to seaweeds have used terrestrial biomass enzymes. For example, the use of cellulases for the release of glucose (fermentable sugars) could result in high glucose yields. However, the terrestrial biomass enzymes show slow enzymatic kinetics and low substrate specificity, and thus, the exploitation of novel enzymes potential from marine sources could improve the extraction efficiency and boost the EAE process [28].

#### 3.2.4. Supercritical Fluid Extraction

Supercritical fluid extraction (SFE) is a technology that mostly uses carbon dioxide (CO_2_) and water (H_2_O) as supercritical solvents. The purpose of SFE is to reach the critical pressure and temperature of the fluid, operating above this critical point, to penetrate rapidly into solid materials and improve extraction process [28,60,63]. Once the supercritical state is reached, the supercritical fluid density is similar to a liquid and the viscosity is low in a range between liquid and gas, facilitating solute mass transfer from the matrix to the solvent [28]. CO_2_ is the most used solvent because its critical point allows labile compounds extraction. There are some other possible supercritical solvents such as methanol, ethanol, methane, ethane, propane, ethylene, propylene and acetone. However, none of the latter meet the principles of “green chemistry and engineering” [28,60]. SFE can be used for analytical purposes, sample preparation, and extraction of target compounds or removal of undesirable products on a larger scale. The main factors of this technology that affect the extraction process are as follows: temperature (40–90 °C), pressure (100–400 bar), type of fluid, materials size and shape, moisture content and solvent flow-rate. Important advantages include: eco-friendliness, non-flammability, low cost, solvent availability, low time consumption, efficient extraction and high selectivity [60]. The most extracted compounds from seaweed and microalgae using SFE method are antioxidants and neutral lipids, as CO_2_ is a good solvent for non-polar target compounds. For more polar compounds, an organic co-solvent, such as ethanol, is needed, and the advantages over other extraction methods are diluted [60]. Low polar seaweed biocompounds extracted with SFE include polyphenols, fatty acids and fucoxanthin and have application in the food and pharmaceutical industries [28,67,68,69,74]. Bogolitsyn et al. [74] showed that fatty acids and polyphenols from brown seaweeds (*Fucus* sp.) were obtained using SFE technology with a content of 20 mg/g while Roh et al. achieved near 49 mg/g of carotenoids from *Undaria* sp., with a co-solvent. The biggest drawback is for mass production, because SFE is considered expensive due to high equipment costs and energy consumption, so further research is essential to uphold its scale-up feasibility [28,60].

#### 3.2.5. Pressurized Solvent Extraction

Pressurized solvent extraction (PSE) (or subcritical water extraction (SWE) when water is the solvent considered), uses temperature and pressure in a range of 50–300 °C and 35–200 bar, respectively. The solvent is heated above its normal pressure and temperature boiling point and below the critical temperature and pressure, remaining in the liquid state. Moreover, some solvent properties such as density, viscosity and surface tension decrease with increasing temperature, enhancing mass transfer and extraction yields with shorter extraction times [28,60,63]. Water is the most used solvent because it does not generate toxic waste, but other solvents can be used [60,63]. Water also has a peculiar behavior: the dielectric constant is decreased with temperature, increasing the affinity towards less polar compounds. The combined high temperature and pressure allows an increase of target compounds desorption from the material, solvents solubility and diffusion into materials, leading to an overall increase extraction kinetics. PSE has therefore several benefits such as high extraction efficiency, less solvent consumption, short extraction time and non-hazardous (for subcritical water) [28,60]. If needed, the extraction of labile compounds has to be carefully designed to be as short as possible, since they could degrade with high temperature and pressure. Though the potential is high, the literature showing seaweed extractions using PSE is scarce. The compounds extracted include phenolics, polysaccharides (fucoidans, alginates, agar) and amino acids with potential application in the food, pharmaceutical and cosmetic industries [28,60]. Saravana et al. [75] has used SWE to extract fucoidans from *Saccharina japonica* with a yield of 13%. SWE applied by Trigueros [82] on protein fractions of *Gelidium sesquipedale* residues showed to be efficient and the work highlighted that the non-polar selectivity is directly proportional to the severity factors of the extraction method. Furthermore, Gomes et al. [87] were able to extract different polysaccharide fractions from red seaweeds with different functionalities depending on the severity factor applied. Medium severity factors allowed to extract structural polysaccharides (agar), without impairing its gelling ability, while harsher severities hydrolyzed the polysaccharides available into oligosaccharides (eventually with prebiotic activity) or monosaccharides that could be used as substrate to fermentative processes.

The scaled-up equipment and maintenance costs are high due to the need for high operating temperature and pressure and the corresponding safety precautions [60].

#### 3.2.6. Electro-Technologies

Extractions using electric effects can be advantageous, bringing new prospects to the processing industry, due to synergistic effects of temperature and electric fields. Pulsed electric field (PEF) and ohmic heating (OH) are examples of promising and attractive electro-technologies extractions [88].

PEF is an eco-friendly technology that enhance cell membranes permeability, due to the occurrence of electroporation caused by the application of very short pulses of very high voltage electric fields. This technology has a wide range of target applications such as microalgae, bacteria, plant tissues, seaweeds, etc., and is currently used to induce stress, to extract several compounds (i.e., proteins, carbohydrates, etc.). In addition to this versatility, PEF treatment has high efficiency, requires low energy and water consumption, does not need chemicals, generates low heat, and is suitable for industry application [62,88]. In spite of that, disadvantages include electrodes oxidation, erosion, fouling and bubbling, difficult and complex temperature control and high costs of power generator [88]. Similarly, to other technologies, PEF application in seaweeds has been scarcely reported. However, some authors believe that PEF has a good potential in integrated seaweed biorefinery approaches, particularly as a pretreatment to disrupt biomass and facilitate extraction using other complementary technologies [62]. Postma et al. [84] studied PEF on the release of water-soluble carbohydrates and proteins from *Ulva lactuca* and obtained the yields of <15% and 15%, respectively and Robin et al. [85] showed a 20% of protein yield from the same seaweed.

OH applies moderate electric field (MEF) to heat a sample. It is a green technology that provides a uniform, fast and precise heating, with high efficiency in energy transfer. This homogeneous and instantaneous transmission of thermal energy and the fast heating rate allow high temperature application in very short periods and enhance cell membrane permeability, providing high quality products where the compounds can be preserved, comparing to conventional extractions [88,89,90]. The heat is generated according to Joule’s law, where the electrodes are in contact with the materials to be heated (e.g., mixture seaweed/solvent), which work as an electric resistance, and an electric current passes through it. This green technology has some parameters dependence, such as a frequency range of 50 to 25.000 Hz, temperature, electric field intensity between 1 and 1000 V/cm which defines the designation of moderate electric field, treatment duration, type and electrical conductivity of material (range of 0.01 to 10 S/m). The main drawbacks of this green process are similar to PEF, because working at low frequencies may induce electrolysis, electrode erosion and leakage of metals to the medium [88,89,90]. Besides the thermal effects, other non-thermal effects have been described, including membrane permeabilization (electroporation of cellular tissues), causing membrane damage and solutes diffusion, and thus facilitating the extraction of bioactive compounds (i.e., phenolics, fatty acids, pigments, etc.) [88,89,90]. Other described advantages of OH include scale-up easiness, color pigments maintenance, lower decrease in the nutritional value, short extraction times, and high yields. Howsoever, the application of OH method in seaweeds is also scarce.

## 4. Integral Use of Different Seaweed Fractions towards Zero Waste

Nowadays, the global biorefinery concept is widely spread and transversally used in the terrestrial biomass exploitation, though it has very low representativeness in seaweed-based industrial processes. As previously said, seaweeds are a rich source of nutritional elements, such as polysaccharides (e.g., agar, carrageenan, alginate, etc.), proteins, lipids, vitamins, polyphenols, pigments and minerals (e.g., potassium, calcium, iron, etc.), which are widely used in food, feed, cosmetic, energy and pharmaceutical industries. Due to their fast growth rate, photosynthetic efficiency, unique compounds, cultivation in saltwater without fertilization, no competition for agricultural land and biomass production, seaweeds are an interesting sustainable feedstock for biorefinery, with economic and environmental advantages, since their wastes generated from biomass processing, still have some biological value. Algae are defined as the 3rd generation feedstock for biofuels production, with zero or low lignin content, not competing with food and feed, with potential to partly replace terrestrial biomass [91,92].

In fact, the interest in seaweed biorefinery started with the idea of solving economic and environmental drawbacks from terrestrial biomass biorefinery. Seaweed biorefinery allows less residues production, resulting in a close to zero waste system, which increments the value of waste fractions, promoting a circular economy approach based on recyclable and reusable wastes. Not only this strategy benefits an increase in resource efficiency, but also, flexibility and diversity, acting on many fields of study and production [91,93,94]. Moreover, the use in biorefineries of seaweed species that cause eutrophication could prevent negative ecological impacts and provide sustainability of coastal environments, and higher social and economic benefits [84,93]. Nevertheless, the financial viability of the seaweed-based biorefineries will determine their commercial success. However, the economic data associated with seaweed based biorefineries is limited, as well as knowledge on the environmental footprint and life cycle assessment, sustainability and scale-up feasibility [28].

The sustainability of seaweed biomass is managed from production, harvesting, processing, packaging, transport and storage. An economically feasible multistage cascading process has an extraction priority based on the seaweed products value: the compounds with highest value should be extracted first, such as bioactive or functional compounds, while compounds considered with lowest value should be extracted after, such as sugars and minerals. However, the order may be changed depending on other factors. For instance, prior extraction of some fractions may also reduce processing problems (e.g., high viscosity) in the extraction of other fractions as well as improving their final quality, due to the removal of possible contaminants or anti-nutrients. Another criterion may be based in mass and energy balances. For instance, if a big fraction may be previously removed without jeopardizing the functionality of the other fractions, it will substantially reduce the volume of biomass to be further processed, with important advantages in overall biorefinery process. For each recovered fraction, it is still essential to perform several sequential steps such as dewatering, concentration and purification. After each step, the waste and leftovers generated are used as raw material for other production processes in a cascading approach, and the leftovers that cannot be used due to their low quality biomass are used for energy production [91].

**Figure 1:**
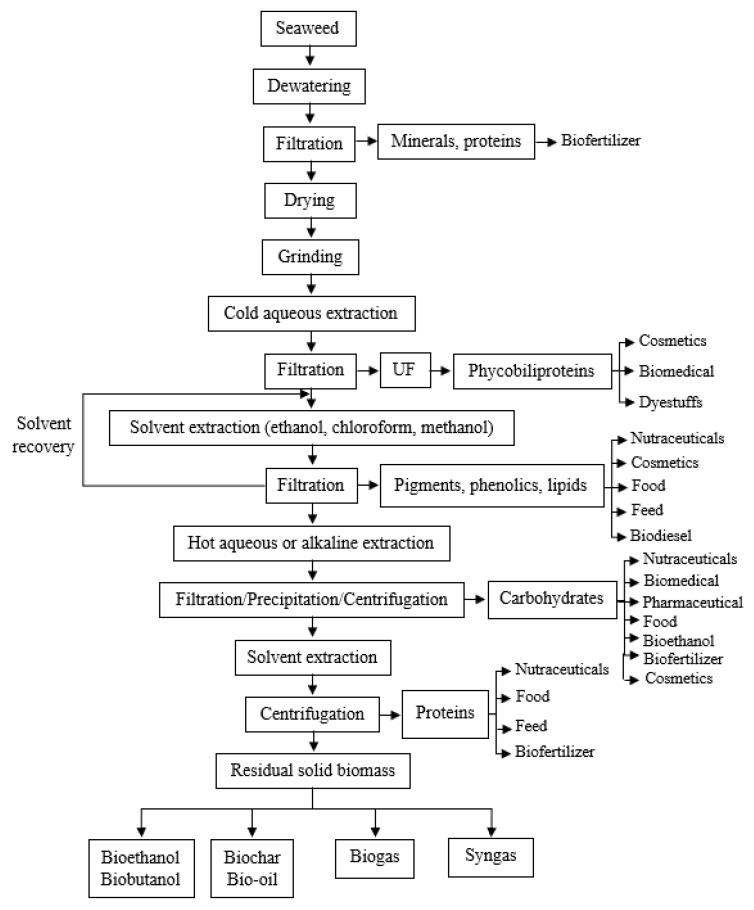
Scheme of seaweed biorefinery approach [87,91,92,93,94,95,96,97,98,99,100].

Seaweeds compounds such as proteins, phenolics, fatty acids and minerals are compounds of interest and preferred for use in a cascade biorefinery due to their variable and high-value applications. Red seaweeds are considered an excellent raw materials to be used in cascade biorefinery [93]. The leftovers, with low value, can be converted to energy (e.g., bioethanol, biogas, biohydrogen, methane, biofuel) or used in other fermentative processes [28,91,93,95]. The final seaweed waste can be further used for different purposes (feed, agricultural and composting). Overall, the efficient use of seaweed biomass is important for the marine environment preservation and organic substances recycling, supporting the circular economy and zero waste concepts [91,95], while reducing the cost of processes. The applications of these sequentially recovered fractions from seaweed biorefineries are similar to those of the individually recovered described in the above sections, including their use as fuels, food ingredients, cosmetics, nutraceuticals, therapeutics and biofertilizers. These products can be readily used in the market or can be used as ingredients or raw materials in other industrial processes to obtain value-added products [94]. A possible general scheme of an integral utilization of different seaweed fractions is proposed in Figure 1.

Biorefinery operates with several sequential processing steps into a single facility, using integral seaweed biomass and fractionating it, producing different products with high value and reducing waste and nutrients loss. A wide range of combinations between processes and technologies (including all the above referred) is available and a careful design of the biorefinery must be made, to allow the efficient recovery of all worthy fractions, causing the least environmental impact, while maintaining the bioactive and functional properties of extracts [28,91,93,94,95,96]. Moreover, a comprehensive biorefinery design must have as primary objective to reach global sustainability by evaluating and optimizing economic, environmental, and social aspects. In this context the use of green extraction technologies, such as MAE, UAE, EAE, SFE, PSE, PEF and OH, is a part of the holistic approach for the biorefinery design. The combination of these methods or choosing some of them as a pretreatment can lead to higher yields of extraction, since the conventional methods do not have the same efficiency when used individually [97]. For instance, PEF has been used as a pre-treatment in the recovery of phycobiliproteins, to disrupt the seaweed structure facilitating water extraction [101]. Considering the biorefinery described as an example in Figure 1, as the functionality of these compounds (e.g., coloring ability) is highly susceptible to heat, PEF is an appropriate non-thermal extraction technology for this first biorefinery step. It is expected that this pretreatment will also facilitates subsequent extraction steps. The color removal (both phycobilliproteins and ethanol soluble pigments) prior to the carbohydrates will allow to valorize these fractions (without thermal degradation) while bleaching and facilitating the subsequent steps. Thermal extraction of carbohydrates can be made either by OH or by PSE with water as solvent [87], allowing energetic advantages and/or tuning for the desired carbohydrate product. Further protein recovery can be made through chemical processes, but also using EAE with proteases and finally the residual biomass can be converted to a glucose rich fraction to be used as fermentation substrate by autohydrolysis (that can be viewed as a particular case of sub-critical water extraction), using high severity factors.

## 5. Food Applications

Raw seaweeds have been consumed worldwide by coastal communities since the beginning of human civilization [26]. They may be eaten in many forms: similar to vegetables, fresh, dried, in flakes, flour or powder, or incorporated in other food products [14,102]. They can be used as a flavoring and seasoning agent, as a condiment, as a snack, as a side-dish or as a whole dish [103]. Their pigments, like carotenoids, phycobiliproteins and chlorophylls, represent a natural source of colorants for the food industry [14]. The first factories of seaweed ingredients appear in 19th century, producing iodine, alginates and carraginates [104]. With the growth on large-scale of food production in the 20th century, there was a widespread use of seaweeds in the industry. In addition, at the period of 1st World War, the seaweeds compounds became to be used for the production of fertilizers, such as potash [9]. The high added-value compounds of seaweeds can be used in bakery, dairy, fish, meat or vegetable-based products allowing nutritional fortification, or the development of new food products with different textures and/or with functional properties [10]. Moreover, seaweeds can be consumed as a substitute of many products, labeled as “fat-free”, “gluten-free”, “mineral rich”, “low sugar”, and “low calories”, or as healthier substitutes of pasta or bacon (*Himanthalia elongata,* as spaghetti, and *Palmaria palmata*, as sea bacon). Seaweeds are ingredients in other recipes such as wraps or in desserts like in innovative Spanish nougats with crushed nori seaweed [14,105]. Moreover, the umami taste of edible seaweeds, can contribute to produce meals with reduced salt, sugar and fat contents, which is of a great importance for a healthy diet [38].

### 5.1. Technological and Nutritional Applications

The seaweeds’ abilities of gelling, thickening and stabilizing are conferred by the hydrocolloids (as agar, alginate, and carrageenan) and have led their extraction and recovery, to be used as texturizing and stabilizing ingredients in the food industry in products like steaks, sausages, pasta, bread, biscuits, smoke cheese, yoghurt or milk deserts, to improve not only their nutritional value, but also their sensory properties [105]. Lately, these unique characteristics have been explored in the innovative industry of molecular gastronomy, creating the famous spheres of gel with flavored liquid inside, helping talented cuisine chefs to achieve the desirable Michelin stars [103].

The first hydrocolloid with European registration, agar, is known as “vegetable gelatin”. It is present in red seaweeds, mainly in *Gracilaria* and *Gelidium* species, and is usually employed in the production of jellies and fruit candies, forming a rigid gel in water at room temperature, making it a high value product for food application [10]. Seaweeds such as *Laminaria japonica* have been used to maintain the product quality for a longer storage period and *Fucus vesiculosus*, as a source of phlorotannins and antioxidant compounds, has found application to prevent food spoilage resultant from oxidative deterioration [106,107]. The alginate extracted from brown seaweeds, such as *Macrocystis*, *Ascophyllum* and *Laminaria*, has been applied for the elaboration of energetic bars, salad dressings, in the preparation of syrups as thickeners and/or emulsifiers, or even to produce ice cream, to avoid the formation of large amounts of ice, and confer a creamy and smooth texture [9]. The sulphated polysaccharides extracted from green seaweeds, the ulvans, have also potential gelling and thickening properties, acting similarly to gum Arabic. Besides that, like agar, ulvans may be used as a vegan alternative for gelatin [26].

Several seaweeds rich in proteins have been used in processing and preparing seafoods, and presenting benefits over traditional high-protein crop in terms of productivity and nutritional value [108]. Some red seaweeds, such as *Porphyra tenera, Porphyra yezoensis* or *Palmaria palmata*, can be highlighted for having a protein content higher than certain legumes such as soybean. Another application for their proteins is as coloring agents potentially useful for food industry, such as the red colored proteins, like phycoerythrin, from red seaweeds. As seaweeds are considered a good protein source for human nutrition, it is possible to use seaweeds as an alternative of animal protein for the processing of foods for athletes that need high consumption of this macronutrient [109].

### 5.2. Functional Foods

The last decades trend of healthy food improved the consumption of seaweeds in Western diets, which was traditionally restricted to Asian and coastal communities [57]. Further the increased interest in health benefits of whole seaweeds led to the elaboration of seaweed-based food products with functional foods claims and promoted the potential application of their bioactive compounds, turning them into lucrative ingredients for industry [110]. Some oligo and polysaccharides extracted from seaweeds are resistant to the digestion process in the upper gastrointestinal tract, presenting potential to be used as prebiotics. This has been confirmed by submitting these compounds to fermentation by fecal microbiota [111]. For instance, besides the technological function already mentioned, carrageenans extracted from *Kappaphycus alvarezii* can act as dietary fiber, clearing the digestive system, protecting the stomach surface membrane and preventing the effects of potential carcinogens on the intestine [112]. One of the most cultivated and commercialized species is *Porphyra* sp., known for their nutritional value with high protein content and a rich amino acid profile with potential to produce cardioprotective effects, antidiabetic, anti-inflammatory and antioxidant properties [113]. Another beneficial effect of seaweed consumption is as a replacer for salt, reducing the risk of chronic diseases provoked by western high NaCl diets, also increasing the consumption of other elements such as calcium, potassium and iodine, usually found in levels below those recommended [107]. The replacement for 1% of seaweeds as *Undaria pinnatifida*, *Porphyra umbilicalis, Palmaria palmata* or *Himanthalia elongata* helped to reduce the salt content up to 50% in frankfurters [114]. Similar results were found replacing the salt in the elaboration of cooked ham, without affecting the yield, texture, color or sensorial acceptance of the reformulated product [115].

## 6. Conclusions

The nutritional and functional profiles of seaweeds are remarkable and their compounds have potential use in a wide diversity of sectors, including the food, pharmaceutical, cosmetics, agriculture and energetic industries.

However, currently this potential is not appropriately explored by the seaweed industry. Extraction processes and conditions to be used have to be thought in a case-by-case approach, considering both the type of seaweed and fractions to be recovered. However, a wide range of possible sustainable technologies and approaches are available to allow effective recovery of different compounds from different biomasses while keeping interesting functional properties. Sustainable practices and zero waste approaches recovering all valuable seaweed compounds in a cascade process are important means to increase seaweed-based industries economic feasibility, with a zero waste generation and reduced environmental impact in line with UN Sustainable Development Goals. These approaches have to be designed holistically, in order to get the highest possible value of the seaweeds’ biomass.

Nevertheless, knowledge regarding seaweed biorefineries is still scarce. Research on alternative extraction technologies, environmental footprint, stocks availability, and scale-up, will be useful to demonstrate the potential and to boost seaweeds application at a commercial scale.

## Figures and Tables

**Table 1:** Different alternative extraction processes of biocompounds from seaweeds.

Seaweed Group	Seaweed Specie	Compounds	Extraction Process	Extraction Conditions	Results	Reference
Brown	*Laminaria japonica*	Carotenoid (Fucoxanthin)	MAE	300 W; 2450 MHz; 60 °C; 10 min; EtOH; 1:15	5.13 mg/100 g	[64]
	*Sargassum fusiforme*				2.12 mg/100 g	
	*Undaria pinnatifida*				109.3 mg/100 g	
Brown	*Laminaria japonica*	Carotenoids	UAE	30 min; MeOH; 1:20	0.336 g/kg	[65]
		Chlorophyll *a*			3.028 g/kg	
Brown	*Undaria pinnatifida*	Carotenoid (Fucoxanthin)	SFE	400 bar; 40 °C; 180 min; 2.32 g/min CO_2_	38.5 mg/g	[66]
				200 bar; 50 °C; 60 min; EtOH; 28.17 g/min CO_2_	0.00753 µg/g	[67]
				400 bar; 60 °C; 270 min; EtOH 1.7–3.2%; 0.005 g/min CO_2_	994.53 µg/g	[68,69]
Brown	*Laminaria japonica*	Carotenoids (Fucoxanthin, lutein, zeaxanthin, *b*-carotene)	PSE	170 bar; 51 °C; EtOH; 10 g/min	0.233 g/kg	[65]
		Chlorophyll *a*			2.335 g/kg	
Brown	*Undaria pinnatifida*	Carotenoid (Fucoxanthin)	PSE	40 bar; 60 °C; DME and EtOH 10%	80%	[70]
				5.9 bar; 25 °C; <43 min; DME; 0.3 g/min	390 µg/g	[68,69]
Brown	*Eisenia bicyclis*	Carotenoid (Fucoxanthin)	PSE	103.4 bar; 110 °C; 5 min; 2 g; EtOH 90%; static	0.42 mg/g	[71]
Brown	*Ascophyllum nodosum*	Total phenolics	UAE	25 min; 0.03 M HCl; 114 µM	143.12 mgGAE/g	[72]
		Fucose			87.06 mg/g	
		Uronic acids			128.54 mg/g	
Brown	*Ecklonia cava*	Fucoidans	EAE	Celluclast; 24 h; 50 °C	1.8%	[73]
Brown	*Fucus vesiculosus*	Fatty acids	SFE	304 bar; 60 °C; 60 min	21.9 mg/g	[74]
		Polyphenols			20.2 mg/g	
Brown	*Saccharina japonica*	Fucoidans	SWE	NaOH 0.1%; 100–180 °C; 100–300 rpm; 20–80 bar; 10–20 min	13.56%	[75]
Brown	*Undaria pinnatifida*	Fucoxanthin	EAE	DME + EtOH co-solvent; pH 6.2; 37 °C; 100 mM; 0.05%	94%	[70]
		Lipids			94%	
Brown	*Carpophyllum flexuosum*	Polyphenols	MAE	1:30; 160 °C; 3 min; 850 W	8.6%	[76]
Red	*Solieria chordalis*	Carrageenan	MAE	KOH 0.5%; 105 °C; 25 min	20%	[77]
Red	*Gracilaria* *vermiculophylla*	Agar	MAE	10–20 min; 70–90 °C	14.4%	[78]
Red	*Gracilariopsis lemaneiformis*	Protein	EAE	6% trypsin; pH 8; 34.6 °C, 6 h	15 mg/mL	[79]
Red	*Porphyra yezoensis*	Peptides	EAE	pH 9; 50 °C; 60 min	55%	[80]
Red	*Hypnea musciformis*	Carrageenan	UAE	500 W; 20 min; H_2_O	49%	[81]
Red	*Gelidium sesquipedale*	Protein	SWE	185 °C; 2 mL/min; 240 min	150 mg/g	[82]
Green	*Ulva intestinalis*	Ulvan	UAE	66 °C; 40 min; pH 7	8.3%	[83]
Green	*Ulva armoricana*	Ulvan Protein	EAE	50 °C, 3 h; 90 °C, 15 min; pH 6	30–40% 2–3%	[61]
Green	*Ulva lactuca*	Protein	PEF	Electric field strength of 7.5 kV/cm with 0.05 ms pulses	15%	[84]
		Carbohydrates			<15%	
Green	*Ulva lactuca*	Protein	PEF	50 pulses; 4 to 6 μs; 50 kV; 0.5 Hz	20%	[85]

Legend: MAE—Microwave-assisted extraction; UAE—Ultrasound-assisted extraction; SFE—Supercritical fluid extraction; PSE—Pressurized solvent extraction; EAE—Enzyme-assisted extraction; SWE—Subcritical water extraction; PEF—Pulsed electric field; GAE—gallic acid equivalent.
